# Comparison of Surgical Techniques Used in Ventricular Septal Defect Closure

**DOI:** 10.21470/1678-9741-2022-0059

**Published:** 2023

**Authors:** Mehmet Çelik, Fatih Aygün, Asım Çağrı Günaydın, Mahmut Gökdemir, Nimet Cindık

**Affiliations:** 1 Department of Cardiovascular Surgery, Konya Application and Research Center, Başkent University, Konya, Turkey; 2 Department of Pediatric Cardiology, Konya Application and Research Center, Başkent University, Konya, Turkey

**Keywords:** Heart Septal Defects, Ventricular, Tricuspid Valve Insufficiency, Echocardiography, Postoperative Period

## Abstract

**Introduction:**

We compared transatrial closure, tricuspid valve septal detachment, and tricuspid valve chordal detachment techniques for ventricular septal defect (VSD) closure.

**Methods:**

Patients who had VSD closure with three different techniques in our clinic between September 2016 and December 2020 were retrospectively reviewed. A total of 117 patients were included in the study. The patients were divided into three groups: group 1, classical transatrial closure; group 2, closure with tricuspid valve septal detachment; and group 3, closure with tricuspid valve chordal detachment. The groups were evaluated by serial transthoracic echocardiography (preoperative, postoperative 1^st^ day, postoperative 1^st^ month). Cardiac rhythm checks and recordings were performed.

**Results:**

No residual VSD was observed in early or late periods in any of the groups whose VSD closure was performed with the three different techniques. No severe tricuspid regurgitation (TR) was detected during the early and late postoperative periods of all operating procedures. When the groups were compared in terms of early/late TR after the operation (without TR+trace amount of TR and mild TR+moderate TR were compared), no statistically significant difference was found (*P*>0,05; *P*=0,969 and *P*>0,05; *P*=0,502).

**Conclusion:**

In this study, we found no statistically significant difference between three VSD closure techniques in terms of early TR, late TR, residual VSD, and permanent atrioventricular complete block during postoperative period. We hope that our results will be supported by the results of researches that are being made about this subject in large series.

## INTRODUCTION

Ventricular septal defect (VSD) closure is the most common congenital heart surgery^[1]^. The definition of a successful VSD closure is the absence of residual VSD, intact tricuspid valve function, and absence of permanent atrioventricular (AV) complete block after the operation. To avoid all of this, VSD rim should be seen clearly. Transatrial VSD closure is the most common VSD closure technique. Nevertheless, in some cases, there are abnormal leaflet tissue or thickened and adherent chordal structures of the tricuspid valve and excessive anterior malalignment of the VSD. All of these can prevent a clear sight of the VSD border. In these cases, tricuspid septal detachment (TSD) and tricuspid chordal detachment (TCD) are used to ensure surgical vision. TSD technique was firstly defined by Hudspeth et al.^[2]^. In this technique, surgical vision is ensured by cutting the septal leaflet from its own annulus. The tricuspid valve leaflet sutured back to its annulus after VSD is closed. Although there were reservations about this technique at first because of prolonged operation and cardiopulmonary bypass (CPB) times, increasing the risk of developing postoperative block and causing dysfunction of the tricuspid valve, publications reporting positive results in the literature are increasing^[3,4,5,6,7,8,9]^. In the TCD method, after VSD is closed with cutting the chordal structure which is blocking surgical vision, it will be implanted in its former place^[10]^. The aim of this study is to compare these three methods in terms of postoperative residual VSD, dysfunction of the tricuspid valve, and permanent dysfunction of the AV node, which are the conditions for a successful VSD closure.

## METHODS

### Patient Selection

Patients whose VSD was closed in our clinic between September 2016 and July 2020 were included in the study. Patients whose VSDs were closed via pulmonary artery, patients whose VSDs were closed via right ventriculotomy, and patients who had muscular VSD were not included in this study. Data were analyzed retrospectively from the hospital database. There was no need for International Review Board approval, consent statement, and clinical trial registration.

### Operative Style and Technique

Before covering with sterile surgical drapes, surgical field was washed with chlorhexidine gluconate (Hibitanol solution, Kim-Pa Ilaç Lab. Tic. Ltd. Sti., Istanbul, Turkey) using warm gauze sponges and then wiped with sterile compresses. Surgical area was washed with povidone-iodine (Poviiodeks Antiseptic solution, Kim-Pa Ilaç Lab. Tic. Ltd. Sti., Istanbul, Turkey). Povidone-iodine was fixed with gauze sponges impregnated with ethyl alcohol (Alkomed®, Istanbul, Turkey). Following sterile coverage, an adhesive surgical sterile drape (Hartmann, Heidenheim, Germany) was applied to the sternum.

After median sternotomy, aorto-bicaval cannulation was performed in all patients. Diastolic arrest was achieved with single-dose antegrade cold crystalloid cardioplegia. Left ventricular vent was placed through the interatrial septum.

VSD was examined transatrially from the tricuspid valve. In cases where surgical vision is adequate, Dacron® patch (Maquet Getinge Group, La Ciotat Cedex, France) or bovine pericardial patch (Edwards Bovine Pericardial Patch, Edwards Lifesciences, Irvine, California, United States of America) was sutured continuously to the VSD with the classical method using 5/0 or 6/0 polypropylene sutures (Propilen®, Dogsan, Trabzon, Turkey).

**Fig. 1:**
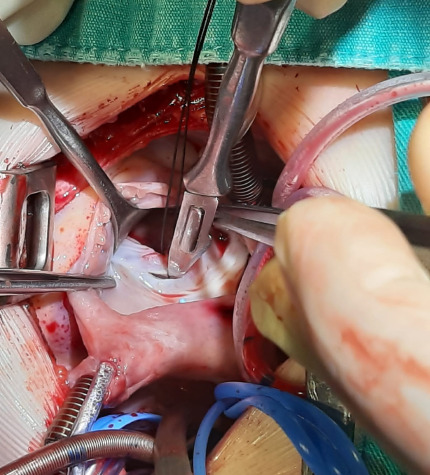
Tricuspid septal detachment.

**Fig. 2:**
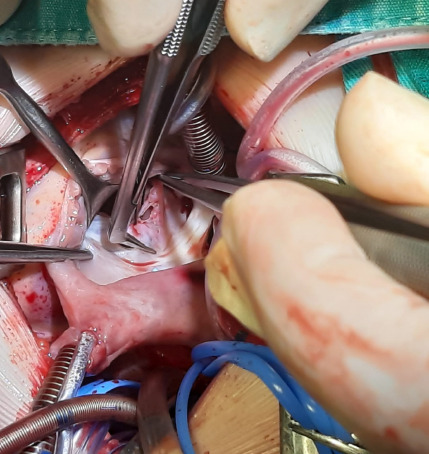
Tricuspid septal detachment.

**Fig. 3:**
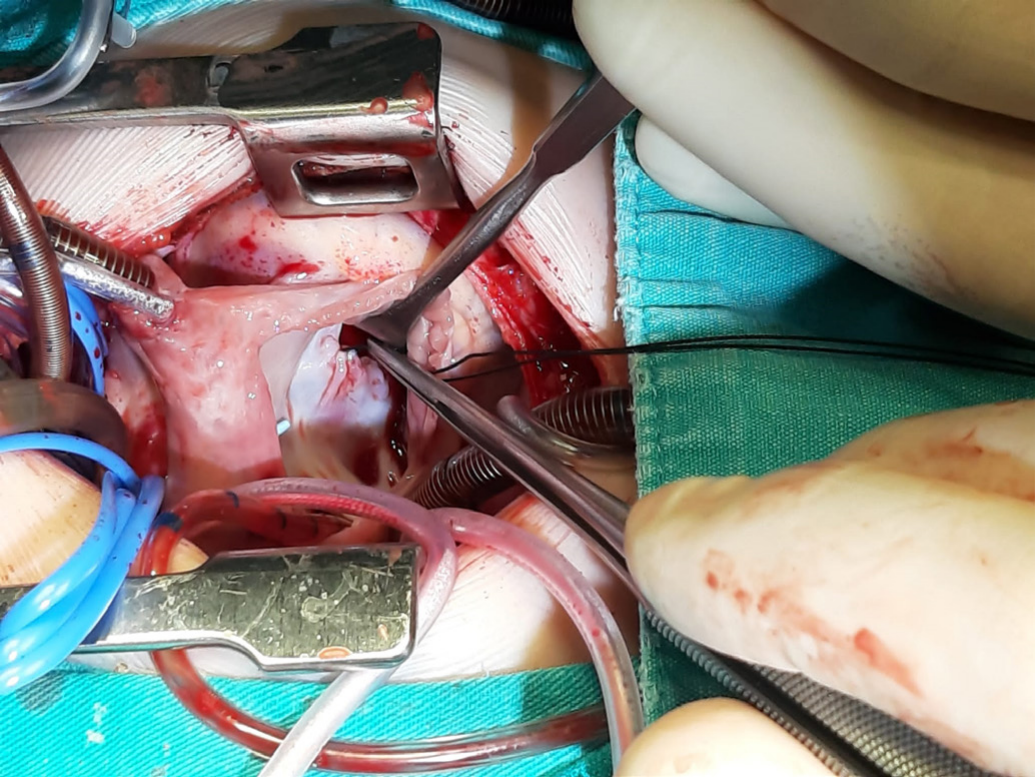
Tricuspid chordal detachment.

Septal leaflet of the tricuspid valve was cut 1 mm from the annulus and parallel to the annulus in the TSD technique (Figures 1 and 2). VSD was closed by suturing the patch continuously with 5/0 or 6/0 polypropylene. Septal leaflet was sutured back to its own annulus primarily in a double row with 6/0 polypropylene sutures. In the TCD technique, the chordae preventing the vision was cut at a distance of 1 mm from the papillary muscle where it was attached (Figure 3). The patch was sutured continuously to the VSD with 5/0 or 6/0 polypropylene. While some of the cut chordae were reimplanted to the papillary muscle where they were cut with 7/0 polypropylene, coaptation sutures were placed with 6/0 polypropylene to the leaflet section where the other chordae were attached. In all three techniques, tricuspid valve insufficiency was checked with cold saline solution after the VSD closure. Simple coaptation sutures were placed if needed.

In all three techniques, weaning protocols from the heart-lung machine and decannulation were routinely applied after VSD closure. After the procedure was completed, the sternum was closed with No. 1 steel wires (Stainless Steel; Ethicon Inc., Somerville, New Jersey, United States of America) using intermittent technique. The subcutaneous tissues were closed with 3/0 braided, absorbable suture (Polysorb, COVIDIEN®, United States of America), and the skin was closed with 4/0 monofilament absorbable suture (Biosyn, COVIDIEN®, United States of America).

### Statistical Analysis

Statistical analyses were performed using SPSS Inc. Released 2009, PASW Statistics for Windows, Version 18.0, Chicago: SPSS Inc. Statistical significance was analyzed by Pearson’s chi-square analysis and when observed values were below the expected values, Fisher’s exact test was used. *P*-value of < 0.05 was considered statistically significant. Control group was not used.

## RESULTS

A total of 117 patients underwent VSD repair. Demographic findings of the patients are given in Table 1. While 57 (47.7%) patients underwent classical transatrial VSD closure repair, the number of patients whose VSD was closed by septal detachment to the tricuspid valve (TSD) was 36 (30.8%) and the number of patients whose VSD was closed by chordal detachment (TCD) was calculated as 24 (20.5%).

**Table 1:** Patients’ demographic and operative data.

	Transatrial closure	TSD	TCD
Number of patients (117)	57 (48.7%)	36 (30.7%)	24 (20.5%)
Age mean (days±SD)	137.5±314	135.1±147.7	145.9±137.8
Weight mean (kg±SD)	5.4±2.6	5.9±2.46	7.5±10.3
Sex (male), 54 (46.1%)	25 (43.9%)	14 (38.9%)	15 (62.5%)
CPB time mean (minutes±SD)	89±37.2	82.9±34.8	69.9±18.6
Cross-clamping time mean (minutes±SD)	56.1±26.6	53.7±21.3	43.2±12
Hypothermia degree mean (°C±SD)	28.8±4.7	31.3±3.2	31.2±1.9
Follow-up period (months±SD)	13±7.1	11.1±6.2	12.5±8.1
Age < 1 month, 7 patients (5.9%)	6 (10.5%)	1 (2.8%)	0
Age 1 - 12 months, 97 patients (82.9%)	46 (80.7%)	30 (83.3%)	21 (87.5%)
Age > 12 months, 13 patients (11.1%)	5 (8.8%)	5 (13.9%)	3 (12.5%)

CPB=cardiopulmonary bypass; SD=standard deviation; TCD=tricuspid chordal detachment; TSD=tricuspid septal detachment

Among the patients included in the study, mean age was found to be 138.5±240 days, and mean weight was found to be 6±5.1 kg; 54 (46.1%) patients were male. Mean CPB time was 83.2±34 minutes, mean cross-clamping time was 52.7±23.1 minutes. While 104 (88.8%) patients were under one year of age, 60 (51.2%) patients’ bodyweight was < 5 kg. The mean follow-up period was 23.4±43.9 months.

In the preoperative echocardiographic data, mild tricuspid insufficiency was found in 37 (31.6%) patients, moderate tricuspid insufficiency was found in 14 (11.9%), and severe tricuspid insufficiency was found in six (5.2%) patients. In 48 patients, there were minimal or no tricuspid insufficiency. None of the patients had postoperative severe tricuspid regurgitation (TR), and no patient had postoperative increased degree of TR. When the groups were compared in terms of early/late TR after the operation (without TR+trace amount of TR and mild TR+moderate TR were compared), no statistically significant difference was found (*P*>0.05; *P*=0.969 and *P*>0.05; *P*=0.502). In any of the groups whose VSD was closed with the three different techniques used, no statistically significant residual VSD (> 2 mm) was observed in the early and late periods. Permanent AV block developed in five patients totally. Three of these were in group 2, one in group 1, and one in group 3. When TCD was compared with the classic method in terms of causing permanent postoperative AV block, no statistically significant difference was found (*P*>0.05; *P*=0.507) (Table 2).

**Table 2:** Postoperative results.

	Transatrial VSD closure (n=57)	Tricuspid septal detachment (n=36)	Tricuspid chordal detachment (n=24)	*P*-value
Early TR				
Without TR	16 (28.1%)	10 (27.8%)	5 (20.8%)	
Trace amount of TR	8 (14%)	6 (16.7%)	5 (20.8%)	0.969*
Mild TR	21 (36.8%)	18 (50%)	10 (41.7%)	
Moderate TR	12 (21.1%)	2 (5.6%)	4 (16.7%)	
Severe TR	0	0	0	
Late TR				
Without TR	26 (45.6%)	16 (4.4%)	6 (25%)	
Trace amount of TR	10 (17.5%)	10 (27.8%)	8 (33.3%)	0.502*
Mild TR	17 (29.8%)	8 (22.2%)	9 (37.5%)	
Moderate TR	4 (7%)	2 (5.6%)	1 (4.2%)	
Severe TR	0	0	0	
Residual early VSD	0	0	0	0
Residual late VSD	0	0	0	0
AV block				
None	46 (80.7%)	29 (80.6%)	22 (91.7%)	
Temporary	10 (17.5%)	4 (11.1%)	1 (4. %)	0.295**
Permanent	1 (1.8%)	3 (8.3%)	1 (4.2%)	0.507***

* Chi-square test result,

** Fisher’s exact test result between transatrial VSD closure and tricuspid septal detachment VSD closure,

*** Fisher’s exact test result between transatrial VSD closure and tricuspid chordal detachment VSD closure AV=atrioventricular; TR=tricuspid regurgitation; VSD=ventricular septal defect

A concomitant procedure was performed in 34 patients included in the study. Major surgical procedures can be sorted as: hypoplastic aortic arch reconstruction on six patients, pulmonary debanding on six patients, right ventricular infundibular resection on six patients, discrete subaortic membrane resection on five patients, double chamber right ventricle repair on three patients, and arterial switch and interrupted aortic arch repair on two patients. Every additional cardiac procedure performed is detailed in Table 3. Even though four patients who underwent additional cardiac surgery had temporary AV node dysfunction, none of them had permanent AV node dysfunction. Also, no significant residual VSD was seen in any of the patients.

**Table 3:** Additional operations with VSD closure.

Surgical Operation	Total (n)	Group 1 (n)	Group 2 (n)	Group 3 (n)
Hypoplastic arcus aorta reconstruction	6	5	1	-
Right ventricular infundibular resection	6	4	2	-
Pulmonary debanding	6	6	-	-
Discrete subaortic membrane resection	5	3	2	-
Double-chamber right ventricular repair	3	3	-	-
Arterial switch operation	2	2	-	-
Interrupted aortic arch repair	2	2	-	-
Vascular ring repair	1	-	1	-
İntracardiac mass excision	1	-	1	-
Pulmonary valvotomy	1	-	-	1
Aortopulmonary window repair	1	1	-	-

VSD=ventricular septal defect

Thirteen (11.1%) patients in the study had genetic anomaly. While 12 patients had Down syndrome, one patient had trisomy 18. Ten of these patients were in group 1, two were in group 2, and one was in group 3. Even though AV dysfunction occurred in two patients from group 1 and the patient from group 3, none of the patients from group 2 had temporary AV node dysfunction. Permanent AV node dysfunction developed in one patient from each the group 1 and 2.

Neither severe tricuspid insufficiency nor significant residual VSD was observed in patients with a syndrome, and there was no perioperative death. Twelve patients in total had already had at least one cardiac intervention before VSD closure surgery. Two patients underwent noncardiac surgical procedures, six patients had pulmonary banding, four patients had balloon angioplasty due to coarctation, one patient had pulmonary banding and aortic coarctation repair, one patient had patent ductus arteriosus ligation, one patient had colostomy, and one patient had surgical procedure due to esophageal atresia.

## DISCUSSION

VSD is one of the most commonly congenital heart anomalies when considered isolated or in association with other congenital cardiac anomalies^[11]^.

Prior conditions for a successful VSD closure are the absence of residual VSD, intact tricuspid valve function, and the absence of permanent AV complete block.

Transatrial VSD closure is the most frequently used and commonly accepted VSD closure technique^[1]^. In addition, in some cases, leaflets of the tricuspid valve and its chordal structures can prevent VSD borders from being well seen. TSD is a surgical technique which is used on such cases, when the entire VSD rim can’t be seen clearly. It was firstly defined by Hudspeth et al.^[2]^. In this technique, surgical exposure is provided by detaching the septal leaflet from its own annulus. The tricuspid valve leaflet is reattached back to its annulus after VSD is closed. There were doubts about this technique. The prolonged operation and CPB times, increasing the risk of developing postoperative block and causing dysfunction of the tricuspid valve, were involved. But the publications reporting favorable results in the literature are increasing.

Pourmoghadam et al.^[5]^ compared TSD and TCD techniques with the classical technique. They stated that both techniques didn’t cause impaired tricuspid valve functions in early and mid-terms. It was notified that the youngest patient age was 87 days, and minimum patient bodyweight was 3.9 kg. Their patients consist of infant and child age group. There were no patients in the study whose VSD was closed during the neonatal period.

Similar to Pourmoghadam, while we included patients who had concomitant atrial septal defect/patent oval foramen closure, patent arterial duct closure, and patients undergoing pulmonary artery interventions, we excluded patients whose VSD was closed via ventriculotomy or pulmonary arteriotomy. But, unlike the other author, we also included patients whose VSD was closed during neonatal period with arterial switch, interrupted aortic arch repair, or hypoplastic aortic arch reconstruction.

In their study, Pourmoghadam et al. stated that CPB time was significantly higher in the TSD group. We didn’t find a significant difference between CPB times in our study. We believe that significant reasons for this conclusion was because we also included patients with long major surgeries such as interrupted aortic arch, arterial switch, and hypoplastic arch reconstruction in our study. We accept the negative outcomes of long CPB times. But we must remember the positive results of a VSD closure surgery in which tricuspid valve functions are preserved without having residual VSD and AV node dysfunction doesn’t develop. Considering the current state of CPB techniques and myocardial protection methods, slightly higher CPB time is an acceptable burden.

Lee et al.^[6]^ compared the classical method with the TSD technique on patients whose body weights are < 5 kg. In their study, they stated that there was no significant difference between both groups in terms of mortality, morbidity, reoperation, residual VSD, aortic valve insufficiency, AV node dysfunction, and tricuspid insufficiency. In addition, they mentioned that when compared with the classical method, progression of tricuspid insufficiency was significantly less in TSD group. A significant portion of our patients (51.2%) also weighed < 5 kg. Similarly, in our study, our results in terms of tricuspid insufficiency, AV node dysfunction, permanent AV block, and aortic valve insufficiency in the relevant patient group were similar with the results of Lee et al. We believe that VSD closure and TVD technique are also safe in early age group.

Bang et al. shared the data of VSD closure surgeries performed with TSD technique on patients younger than three months^[7]^. They applied TVD to 49 (16.6%) of 296 patients in total. They included patients with isolated VSD closure but excluded patients with concomitant cardiac surgeries. They mentioned that CPB times and cross-clamping times were found to be significantly higher in the TVD group compared to the transatrial closure group. In our patient group, no significant difference was found in terms of CPB and cross-clamping times. We think the reason of this is that all three groups included patients with concomitant surgical interventions. They also mentioned that they had to reoperate three patients due to residual VSD and they implanted pacemaker on two (0.6%) patients due to permanent AV node dysfunction. While we have patients younger than three months, we didn’t set an age limit and also didn’t exclude patients with additional cardiac interventions. While according to Bang’s study, permanent AV block development rate was coherent with literature (< 1%), permanent AV block (4.2%) was more common in our patients^[10]^. But there was no need for reoperation due to residual VSD in any of our patients. As the reason for this, we think that our efort for not leaving a residual VSD may contribute to the development of permanent AV block higher than normal.

It has been reported in the literature that approximately 20-30% of all VSDs require TSD^[3]^. In our study, TVD and TCD techniques were applied on 60 (51.2%) patients in total. According to this, we can say that we use TVD and TCD techniques more often. We believe reasons for this are both techniques adequately improve surgica vision, both are easily applied, they don’t take much time, and they also don’t impair tricuspid valve functions. When compared to the iterature, development of AV block rate is higher in our study. We think that this happened because of excessive traction due to the efort not to leave residual VSD during learning curve period. Although, according to literature, these techniques are commonly used, TSD and TCD should be avoided unless they are necessary due to the fragile and sensitive tricuspid valve structures during neonatal period.

### Limitations

The number of patients is strict. We tried to handle that with a long study time, lasting four years. We know that the Pediatric Cardiologist is the most important eye in postoperative cardiac echocardiographic examination. Our cardiologists were talented and cooperative.

## CONCLUSİON

For a successful VSD closure, there should be no residual leakage, no AV block development, and tricuspid valve structure and functions should be preserved. And to achieve this, a surgical vision in which VSD borders can be clearly seen should be provided. If there is a imited surgical exposure of the VSD, we believe that VSD closure with TSD and TCD methods are safe.
